# Higher Age (≥60 Years) Increases the Risk for Adverse Events during Autologous Hematopoietic Stem Cell Transplantation

**DOI:** 10.3390/cancers15051584

**Published:** 2023-03-03

**Authors:** Monika Haubitz, Vittoria S. von Petersdorff, Ingrid Helsen, Claudio Brunold, Elisabeth Oppliger Leibundgut, Gabriela M. Baerlocher

**Affiliations:** 1 Laboratory for Hematopoiesis and Molecular Genetics, Experimental Hematology, Department for BioMedical Research (DBMR), University of Bern, 3008 Bern, Switzerland; 2 Department of Hematology, Inselspital, Bern University Hospital, University of Bern, 3010 Bern, Switzerland

**Keywords:** autologous hematopoietic stem cell transplantation, transplant product, hematopoietic stem and progenitor cells, adverse events, adverse reactions, collection, processing, transplantation, age

## Abstract

**Simple Summary:**

Autologous hematopoietic stem cell transplantation (autoHSCT) is a highly regulated procedure for the treatment of various diseases. In a retrospective, observational study from adult patients treated with autoHSCT during the years 2016–2019, we evaluated the occurrence and severity of adverse events (AEs) related to each step from collection to infusion and investigated whether certain factors correlated with the occurrence and number of AEs. Of the 449 patients, 19.6% had AEs, and only 6.0% experienced adverse reactions (ARs); 25.8% of the AEs were serious and 57.5% were potentially serious. Larger leukapheresis volumes, lower numbers of collected CD34+ cells, and larger transplant volumes significantly correlated with the occurrence and number of AEs. Moreover, patients over 60 years experienced significantly more AEs. By preventing AEs related to quality and procedure, potentially serious AEs could be reduced by 36.7%. These results highlight potential steps for optimization of the autoHSCT procedure, especially in elderly patients.

**Abstract:**

Autologous hematopoietic stem cell transplantation (autoHSCT) is a standard of care for patients with hemato-oncologic diseases. This procedure is highly regulated, and a quality assurance system needs to be in place. Deviations from defined processes and outcomes are reported as adverse events (AEs: any untoward medical occurrence temporally associated with an intervention that may or may not have a causal relationship), including adverse reactions (ARs: a response to a medicinal product which is noxious and unintended). Only a few reports on AEs cover the procedure of autoHSCT from collection until infusion. Our aim was to investigate the occurrence and severity of AEs in a large data set of patients who were treated by autoHSCT. In this retrospective, observational, single-center study on 449 adult patients during the years 2016–2019, AEs occurred in 19.6% of the patients. However, only 6.0% of patients had ARs, which is a low rate compared to the percentages (13.5–56.9%) found in other studies; 25.8% of the AEs were serious and 57.5% were potentially serious. Larger leukapheresis volumes, lower numbers of collected CD34+ cells and larger transplant volumes significantly correlated with the occurrence and number of AEs. Importantly, we found more AEs in patients >60 years (see graphical abstract). By preventing potentially serious AEs of quality and procedural issues, AEs could be reduced by 36.7%. Our results provide a broad view on AEs and point out steps and parameters for the potential optimization of the autoHSCT procedure, especially in elderly patients.

## 1. Introduction

Autologous peripheral hematopoietic stem and progenitor cell (HSPC) transplantation (autoHSCT) is routinely used for the treatment of hematological diseases such as plasma cell disorders (PCD), non-Hodgkin lymphoma (NHL), and acute myeloid leukemia (AML) [1,2,3]. The process of autoHSCT, including the phases of cell collection, processing, storage, transportation, and administration, is highly regulated by the international standards FACT-JACIE (FACT: the Foundation for the Accreditation of Cellular Therapy)-(JACIE: the Joint Accreditation Committee of International Society for Cell and Gene Therapy (ISCT) and the European Society for Blood and Marrow Transplantation (EBMT)) [4]. 

Deviations from the standard procedures called adverse events (AEs), including adverse reactions (ARs), in patients during or after infusion of the transplant must be reported, and short- and long-term corrective and preventive actions must be defined. FACT-JACIE defines AEs as “any unintended or unfavorable sign, symptom, abnormality, or condition temporally associated with an intervention that may or may not have a causal relationship with the intervention, medical treatment, or procedure”. AEs might be sub-classified into activity steps (e.g., procurement, testing, transport, processing, storage, distribution) and in subcategories (tissue or cell defect, equipment failure, materials, system failure, human error, others) [5]. In addition, such AEs are graded into minor, moderate or major severity [4]. ARs, a type of AE, are defined as “a noxious and unintended response suspected or demonstrated to be caused by the collection or administration of a cellular therapy product or by the product itself” [4]. These typically range from mild reactions such as nausea, vomiting, chills, tremor, headache, numbness in extremities, stomachache, bad smell, dizziness, diarrhea, flushing, fever, and cough to life-threatening reactions affecting the cardiovascular, respiratory, and neurological systems [6]. 

Transplant-infusion-related ARs are commonly attributed to dimethyl sulfoxide (DMSO) toxicity as well as to toxicity of plasma or red blood cell components, especially those described in allogeneic stem cell transplants [7,8]. DMSO is required as a protective agent to maintain cell viability during the process of cryopreservation and thawing of the cellular transplant product. Reduction of the DMSO concentration from 10% to 5% resulted in improved CD34+ cell viability, improved clonogenicity of cells, and lower rates of ARs [9,10,11,12], as reviewed in Kollerup Madsen [7]. High numbers of leukocytes (Lc), especially polynuclear cells (PNC) and mononuclear cells (MNC), have been associated with increased frequency of ARs [13,14,15,16,17,18]. Other risk factors include patient age, red blood cell count, and larger infusion volumes [6,19,20,21]. In addition, Bojanic et al. identified female gender and diagnosis of multiple myeloma as significant predictors of ARs during infusion of the transplant [13].

Apart from the above-described risk factors for ARs, other deviations from the standard operating procedures (SOP) occurring during the entire procedure of autoHSCT may influence transplant quality as well as outcome, morbidity, and mortality. While ARs after autologous or allogeneic infusion of HSPCs have been extensively investigated, hardly any studies reported AEs during the process of autoHSCT from the collection of HSPCs until the infusion of the cellular product. The aim of our study was to assess the incidence and grade of AEs occurring during the entire procedure and to identify potential targets for improving the performance of the autoHSCT procedure.

## 2. Patients and Methods

### 2.1. Patients and autoHSCT

In this retrospective, single-center, observational study, data from 449 patients who had undergone autoHSCT at the University Hospital Bern from 2016 to 2019 were evaluated. Only adult patients (age ≥ 18 years) who had a collection of HSPC and one or more autologous HSPC infusions and had signed the informed consent were included. Patients underwent disease-specific mobilization regimens, including chemotherapy plus granulocyte-colony stimulating factor (G-CSF) at a dose of 5–10 µg/kg/day or G-CSF alone by subcutaneous injection. The HSPC collection was started when the peripheral blood CD34+ cell count was higher than 10–20 × 10^6^/L. Large-volume leukapheresis was performed with either the COBE Spectra (COBE) (TerumoBCT) or the Spectra Optia MNC (Optia) (TerumoBCT) cell separators with a mononuclear collection protocol. The target dose of CD34+ cells was defined individually for each patient dependent on the number of transplants aimed for and on the transplant physician’s decision for a CD34+ cell selection. At least a total of CD34+ cells > 2 × 10^6^/body weight (BW)/transplant were collected. The apheresis cell product was either processed immediately after collection or stored overnight at 4 °C and processed the following morning. 

Transplant products were minimally processed by volume and/or platelet reduction and cryopreserved at a cell concentration up to 2 × 10^8^ cells/mL with 5% DMSO (controlled rate freezing and storage below −165 °C in the vapor phase of liquid nitrogen). For some transplant products, a CD34+ cell selection (positive selection) was performed to reduce the transplant volume and/or the potential of contamination with malignant cells. 

The cryopreserved transplant products were thawed in a 37 °C water bath, transferred to 50 mL syringes (10 mL syringes for selected material) under sterile conditions, and infused in the patient within an hour. The volume of the transplant product was determined by the CD34+ cell dose requested by the transplant physician, typically 3–5 × 10^6^/BW/transplant; however, in general, it did not exceed 280 mL per day, otherwise it was fractionated up to 5 days (several infusions for one transplantation). Patients received pre-infusion medication with antihistamines and corticosteroids. Vital signs were monitored every 15 min during and for 1–2 h after the infusion. 

### 2.2. Parameters for Analyses

Most parameters analyzed from patients undergoing autoHSCT at our center were extracted from a custom based informatics application and database that was built up over the last years in order to optimize workflows and data collection, secure critical steps, as well as improve quality. For each autoHSCT, the following parameters were collected: gender, age at transplantation, type of disease, total volume of collected cellular product, and blood values at first leukapheresis (including total Lc, MNC, PNC, and CD34+ cell counts per transplant product as well as eventual performance of CD34+ cell selection). In addition, the infused volume of the transplant product and DMSO content, the body temperature during and after infusion of the transplant product, the number of infusions and transplantations, as well as several outcome parameters during the hospitalization (time to absolute neutrophil count of >0.5 × 10^9^/L and platelet count of >20 × 10^9^/L, body temperature > 38.5 °C, and duration of hospitalization) were assessed. Since a CD34+ cell selection requires higher cell numbers to be collected, influences the cellular composition of the transplant product, and leads to much lower transplant volumes and lower DMSO volume per transplantation, data from patients with a CD34+ cell selection were excluded from certain statistical analyses. 

### 2.3. AEs

Every deviation from the SOP during the process of autoHSCT from collection of HSPCs until the infusion of the autologous transplant product was registered as an AE. AEs were categorized according to the part of the transplantation process, e.g., collection, processing, and infusion. AEs which could not be assigned to a patient were categorized as non-patient-assigned AEs. Furthermore, AEs were subcategorized into issues of administration, procedure, quality, material, infrastructure, ARs (defined as event on the day of cellular infusion), and these were graded into “serious”, “potentially serious”, and “non-serious” according to the definitions of FACT-JACIE and the EU directive [4,5,22,23]. “Serious” was defined as “any untoward occurrence associated with the procurement, testing, processing, storage and distribution of tissues and cells that might lead to the transmission of a communicable disease, to death or life-threatening, disabling or incapacitating conditions for patients or which might result in, or prolong, hospitalization or morbidity” and “non-serious” as “any AE not classified as serious” and as “any AEs that causes interference with routine daily activities without major discomfort and these interferences do not persist” [5,23,24]. For our purposes, we also introduced “potentially serious” and defined it as any AEs that cause interference with routine daily activities with a great potential for a major discomfort and persistence of the interference if no immediate measures had been taken. 

### 2.4. Statistics

Anonymized data were used for descriptive and inferential statistics (T-, Chi-square and Fisher’s exact test) as appropriate to the data. T-tests were applied to compare data of two groups, and in the case of neutrophil and platelet counts, ranked data were used for analysis (patients who never reached the minimum value were set in the last rank and the ones who never had a value below the limit were set in the first rank). *p*-values < 0.05 were considered statistically significant. 

## 3. Results

### 3.1. Characteristics of Patients and Transplants

**Figure 1:**
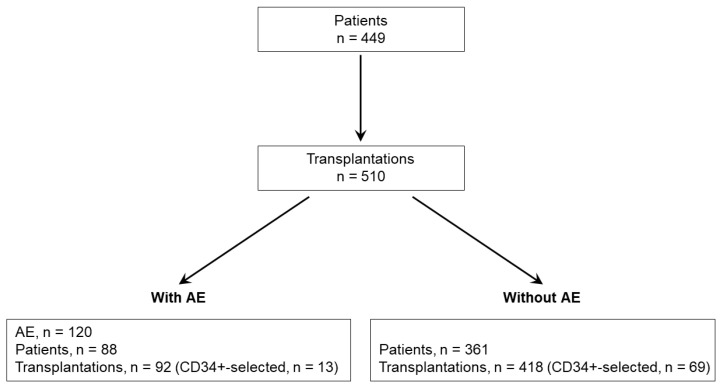
Flow chart of the patient cohorts and transplantations with or without AEs.

A total of 449 patients who underwent autoHSCT with 510 transplantations were included in the study (Figure 1); 305 (68%) patients were males and 144 (32%) females (Table 1). The age at first transplantation ranged from 19 to 79 years with a median of 60 years, and 208 (46%) patients suffered from PCD, 164 (37%) from NHL, 44 (10%) from AML, and 33 (7%) from other diseases (e.g., germ cell tumor or Hodgkin lymphoma). 

Most patients received 1 transplantation (*n* = 401, 89%), 35 (8%) received 2 transplantations, and 13 (3%) received 3 separate transplantations (Table 2). 339 (85%) patients received 1 infusion per transplantation, including 80 (24%) patients with CD34+-selected transplants. In detail, 30 (86%) patients with 2 transplantations received 1 infusion per transplantation, including 2 patients with CD34+-selected transplants, and 5 patients with 2 transplantations received 2 or more infusions per transplantation (range 2–5); 13 patients had 3 transplantations with 1 infusion each.

At the day of the first leukapheresis, patients (excluding the ones with CD34+-selection) had a hemoglobin of 112 g/L, a Lc count of 5.3 × 10^9^/L, and a platelet count of 225 × 10^9^/L. The volume of the leukapheresis product was 287 mL (range 89–700 mL), containing a median of 8.7 × 10^6^/kg BW of CD34+ cells (Table 3). The composition of the leukapheresis consisted of a Lc count of 200 × 10^9^/L with MNC count of 114 × 10^9^/L and a PNC count of 86 × 10^9^/L. A median volume of 220 mL was infused per transplantation with a 5% DMSO content; with higher volumes, infusions were split over 2–5 days to reduce the daily infusion volume and especially the volume of DMSO per day. Hence, a median dose of 11 mL DMSO was applied per transplantation (range 2–84 mL). After transplantation, the median time to neutrophil and platelet recovery was 11 days and 14 days, respectively; 401 (94%) patients experienced a body temperature ≥ 38.5 °C at some time after transplantation, and the median duration of hospitalization was 21 days (range 1–71 days). 

### 3.2. Adverse Events (AEs)

A total of 120 AEs were reported in 510 transplantations (Figure 1), with 109 (91%) AEs occurring in 88 patients with 92 transplantations and 11 AEs being non-patient assigned, while 361 patients with 418 transplantations had no AEs; 13 (14.8%) patients with AEs and 69 (19%) patients without AEs received CD34+-selected transplants, and 28 (23.3%) of patient-related AEs were ARs. 

**Figure 2:**
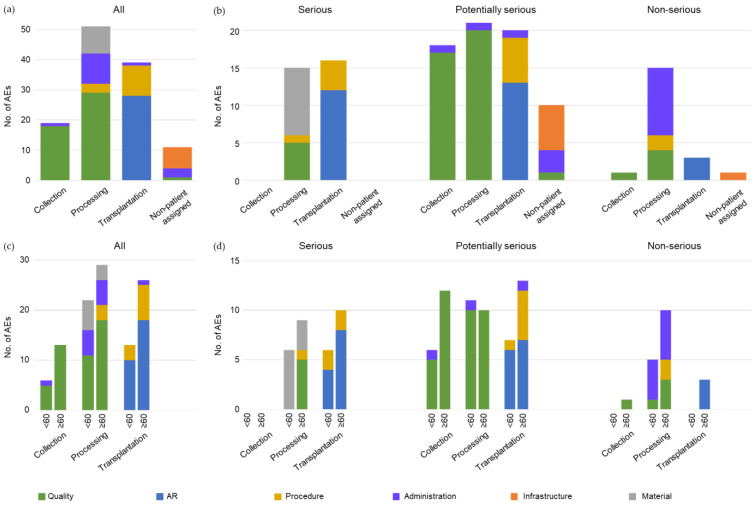
AEs per category (collection, processing, transplantation, non-patient assigned) and subcategory (quality, AR, procedure, administration, infrastructure, material). (**a**) All AEs, (**b**) graded according to seriousness, (**c**) according to age (transplantations in patients <60 years, *n* = 256 and ≥60 years, *n* = 254), and (**d**) according to age and seriousness.

Overall, 19.6% of all patients experienced AEs, and 6.0% of all patients had an AR (one patient with two ARs); 42.5% of AEs occurred during processing, 32.5% during transplantation, 15.8% were reported during collection, and 9.2% were non-patient assigned (Figure 2a). Moreover, 25.8% of AEs were classified as serious, 57.5% were potentially serious, and 16.7% were non-serious (Figure 2b). Quality issues were by far the most prevalent AEs during collection and processing (39.2%), whereas ARs were the most frequent events during transplantation, accounting for 23.3% (*n* = 28) of all AEs. 

Serious AEs reported during processing were mainly related to material (e.g., damaged freezing bag) and quality (e.g., aggregates in the transplant product), while most serious events during transplantation were ARs or events related to procedure (e.g., time delays) (Figure 2b; Supplemental Table S1). Potentially serious AEs occurred at similar rates in the three categories of the autoHSCT process. During collection and processing, potentially serious AEs almost exclusively consisted of quality issues (e.g., cell loss over defined limit, insufficient cell collection), and events during transplantation were either ARs or related to procedure (e.g., time delays). Most non-serious events occurred during processing and were mainly related to administration (e.g., incorrect data entry). 

### 3.3. Transplantations with AEs

Patients with AEs (with AEs) were significantly older at transplantation than patients without AEs (without AEs) (median age 62 vs. 58 years, *p* = 0.0013; graphical abstract). Patient laboratory values at the start of HSPC collection and cellular composition of the transplant were not significantly different in patients with AEs compared to patients without AEs (Table 3). The median number of CD34+ cells collected during leukapheresis was significantly lower in patients with AEs than in patients without AEs (6.2 vs. 9.6 × 10^6^/kg BW, *p* < 0.0001), and the median leukapheresis volume was significantly larger in patients with AEs (374 mL vs. 273 mL, *p* < 0.0001). Furthermore, the median volume transplanted was significantly larger in patients with AEs (310 mL vs. 180 mL, *p* < 0.0001), with, accordingly, a significantly larger median volume of DMSO of 15.5 mL vs. 9.0 mL per transplantation in patients with AEs compared to patients without AEs (*p* < 0.0001). The comparisons were still statistically significant when we analyzed subgroups of patients with patient-related (ARs and insufficient collection) and patient unrelated AEs. Patients with AEs needed significantly more time for neutrophil and platelet recovery (*p* = 0.0182 and 0.0120), and the duration of hospitalization was slightly but significantly longer for patients with AEs than for those without AEs (22 vs. 21 days, *p* = 0.0436). No significant differences were found for body temperature > 38.5 °C, positive blood cultures, and infections caused by a catheter when comparing the cohorts with AEs and without AEs. 

### 3.4. AEs and Age at Transplantation

Patients ≥ 60 years of age had significantly more AEs than younger patients (26.8% vs. 16.0%, *p* = 0.017, graphical abstract). This difference applied to all steps of autoHSCT including collection, processing, and transplantation (Figure 2c). Patients ≥ 60 years experienced more quality issues during collection and processing than younger patients, especially serious ones, i.e., collection of fewer cells than targeted, insufficient cells collected, and inadequate cell loss during processing (Supplemental Table S1). Likewise, AEs occurring during transplantation concerning the procedure (e.g., time delays) were more frequent in older patients; however, these were mostly only potentially serious (Figure 2d). There were also slightly more ARs in patients ≥60 years.

**Figure 3:**
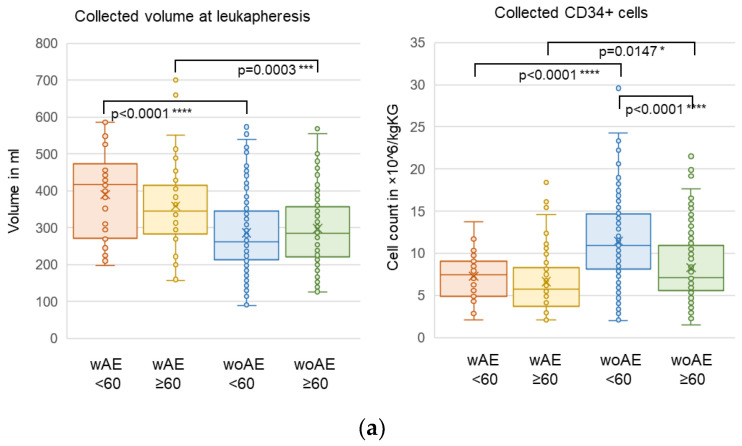

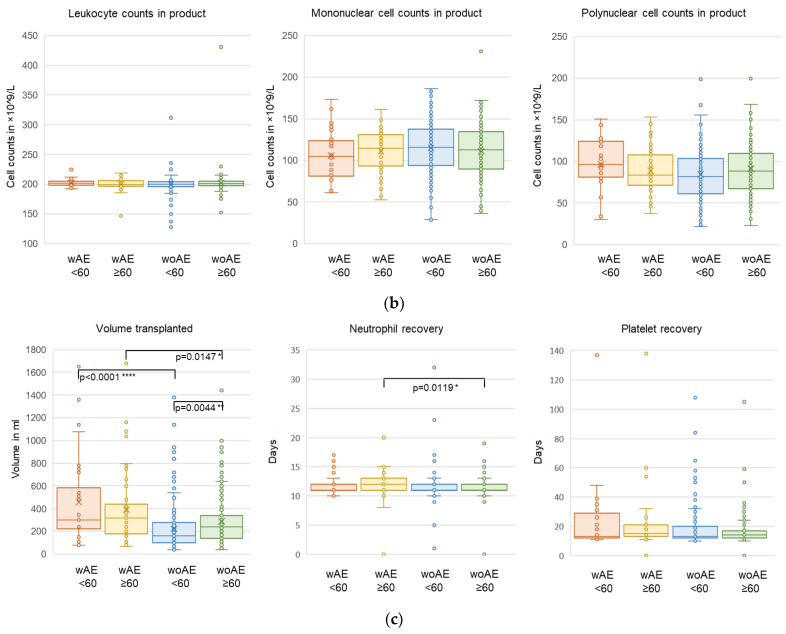
Parameters according to AEs and age. (**a**) Collected volumes and CD34+ cells during leukapheresis, (**b**) numbers of Lc, MNC, and PNC in the transplant product, (**c**) total transplanted volumes and times to reach an absolute neutrophil count of >0.5 × 10^9^/L and a platelet count of >20 × 10^9^/L. Data without the CD34+-selected transplants; significance: * *p* ≤ 0.05, ** *p* ≤ 0.01, *** *p* ≤ 0.001, **** *p* ≤ 0.0001.

The significant differences in collected CD34+ cells, leukapheresis volumes, and transplant volumes observed in the whole cohort were present in both the younger and older age groups (Figure 3a,c, Supplemental Table S2). There were no differences in number or subtypes of Lc in the transplant products (Figure 3b). Of note, patients ≥ 60 years with AEs needed significantly more time to reach neutrophil recovery than patients without AEs (median 12 vs. 11 days; *p* = 0.0119, using ranked data), but no significant difference was seen in patients < 60 years, and platelet recovery time was not different among groups (Figure 3c). 

A rather strong Pearson correlation (ρ ≥ |+/−0.5|) was found for the volume collected at leukapheresis and transplanted (ρ = 0.55), for the collected and transplanted CD34+ cells (ρ = 0.57), as well as for the transplantation volume and number of days to infuse the transplant (ρ = 0.88), indicating that these factors are not independent. Among the four groups, collected CD34+ cells, Lc, MNC, and PNC counts in the transplant product, and transplanted volumes did not significantly differ between males and females.

### 3.5. Number of AEs per Transplantation 

**Figure 4:**
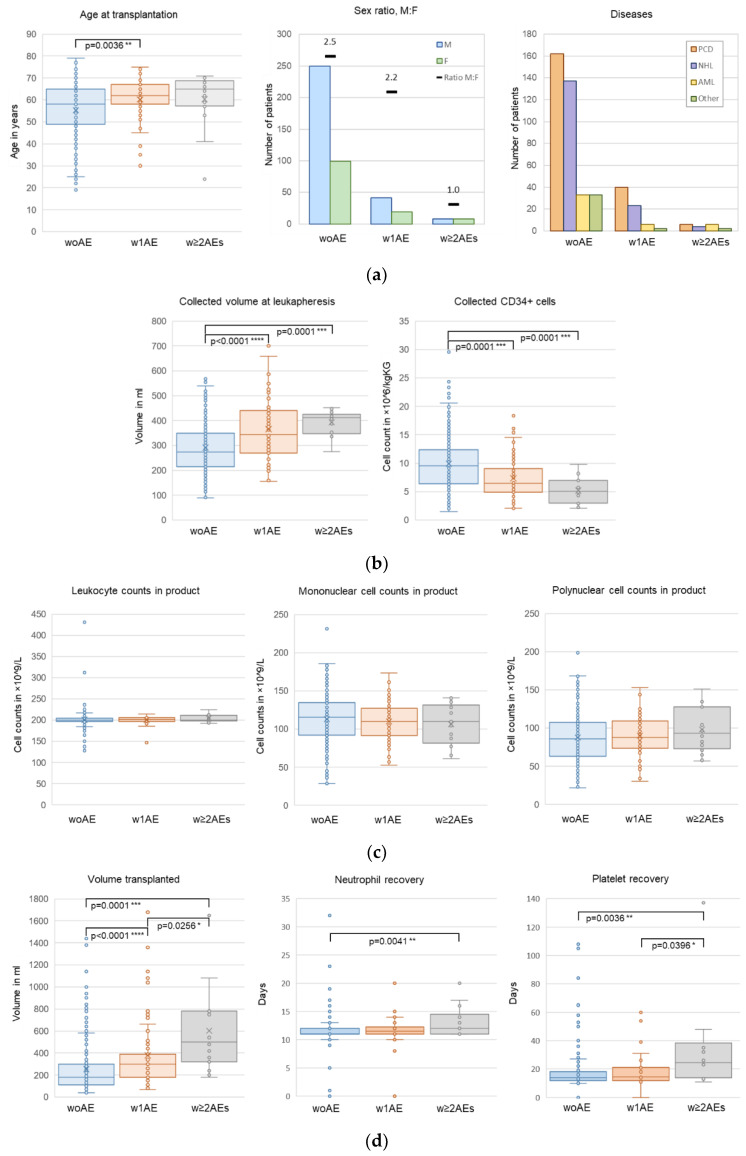
Parameters according to number of AEs. (**a**) Age, sex, and disease (all data included), (**b**) collected volumes and CD34+ cells during leukapheresis, (**c**) numbers of Lc, MNC, and PNC in the transplant product, (**d**) total transplanted volumes and times to reach an absolute neutrophil count of 0.5 × 10^9^/L and a platelet count of >20 × 10^9^/L. Data without the CD34+-selected transplants; significance: * *p* ≤ 0.05, ** *p* ≤ 0.01, *** *p* ≤ 0.001, **** *p* ≤ 0.0001.

In total, 1 AE was reported in 63 transplantations (w1AE) and two or more AEs (w ≥ 2AEs) in 16 transplantations (range 2–4). Patients w1AE were significantly older at transplantation than patients without AEs (62 vs. 58 years, *p* = 0.0036), and patients w ≥ 2AEs were even older with a median age of 65 years (Figure 4a). In addition, the number of AEs correlated with a relative increase of females (ratio male to female: without AEs, 2.5; w1AE, 2.2; w ≥ 2AEs 1.0). The distribution of disorders (PCD, NHL, AML) was equal among groups. Low CD34+ cell counts, high leukapheresis volumes, and high infusion volumes significantly correlated with the number of AEs (Figure 4b,c, Supplemental Table S3). Again, no significant differences were observed in Lc, MNC, or PNC counts of the transplant product. Patients w ≥ 2AEs received significantly higher volumes per transplantation (500 vs. 180 mL; *p* = 0.0001) and needed significantly longer to reach neutrophil recovery (12 vs. 11 days; *p* = 0.0041) and platelet recovery than patients without AEs (24.5 vs. 14.5 vs. 14 days; *p* = 0.0396 and *p* = 0.0036) (Figure 4d).

## 4. Discussion

Performing autoHSCT in Switzerland, a standard of care for patients with hemato-oncologic diseases, and being reimbursed for this procedure necessitates a quality assurance system and adherence to regulations by FACT-JACIE. While ARs after autologous or allogeneic infusion of HSPCs have been extensively investigated, hardly any studies assessed AEs during the different processes of autoHSCT. In our retrospective, observational, single-center study, we investigated the incidence and grade of all reported deviations from SOPs starting with the collection to infusion of the transplant as defined in the standards by FACT-JACIE, and we identified potential targets to improve the performance of the autoHSCT procedure [4]. 

Our patient cohort had a median age of 60 years, which was in the range reported in recent studies (56–59 years) [20,21,25]. In contrast, patient cohorts in earlier studies had a lower median age of 42–51 years [6,13,15,16]. This difference reflects how the selection of patients for autoHSCT has shifted towards higher age. Nowadays, transplant eligibility is based on biology and performance status of the patient rather than chronological age, and the procedure is considered safe and effective even for patients in their seventies [26,27,28,29]. 

Overall, 19.6% of our patients experienced one or more AE, which is a low rate compared to other reports. We observed a particularly low incidence of 6.0% for ARs compared to rates of 13.5–56.9% reported in five comparable patient cohorts [13,14,16,20,25]. In four of these cohorts, the transplant product was cryopreserved with 10% DMSO and infused without DMSO reduction [13,16,20,25]. In the study with the lowest rate of ARs (13.5%), the transplant was washed before infusion in order to reduce DMSO concentration [14]. The beneficial effect of DMSO reduction to 5% was confirmed in several studies [9,10,30]. Apart from the DMSO concentration, it has been reported that high PNC counts in the transplant product led to higher numbers of ARs, probably due to increased numbers of clumped cells and debris, whereas others did not find this correlation [16,31]. In our study, Lc, MNC, and PNC counts per volume did not correlate with the occurrence of ARs and AEs. A reason for the low incidence of ARs could also be the premedication given to all patients before infusion of the cellular product.

Our data reveal significant correlations of AEs with both low CD34+ counts, large volumes of apheresis products, and large transplant volumes, including larger volumes of DMSO in age groups over and below 60 years. Interestingly, these parameters also significantly correlated with the number of AEs. Mobilization of CD34+ cells from the bone marrow into the peripheral blood is influenced by several factors such as increasing age, prior treatment, and genetics [32]. In more detail, mobilization failure of autologous donors can correlate with the number of prior lines of chemotherapy treatment due to toxicity on HSPCs as well as the bone marrow niche [33]. Due to the retrospective nature of the study, we were not able to systematically assess all prior treatments and the genetics of each patient. Poor mobilization is often found in patients >60 years of age based on “age-related senescence” of HSCs due to progressive telomere shortening [34]. Telomere attrition is one of the hallmarks in aging HSCs of mammals and humans [35,36,37,38]. In addition, shortening of telomeres has been found in recipients of autologous and allogeneic hematopoietic stem cell transplantation [39,40]. As low CD34+ counts require larger apheresis volumes to reach the critical cell count needed for transplantation, larger transplant volumes must be infused, and larger volumes of DMSO may lead to higher toxicity. Therefore, splitting of infusions over several days might increase tolerability and reduce toxicity. In addition, we observed a trend regarding lower numbers of CD34+ cells collected in females compared to males in patients without AEs < 60, but no significant gender difference was seen for Lc, MNC, or PNC counts. Similarly, Zhang et al. reported fewer CD34+ cells collected in females compared to males and no difference for MNC counts [41].

Of interest, we found significantly more AEs in patients over 60 years, and this age group comprised a higher number of patients with more than one AE. Moreover, patients ≥60 years had more serious ARs than the younger age group. This difference might reflect potential complications due to the higher number of comorbidities in older patients. Comorbidities such as cardio- and neurovascular diseases associated with aging, reduced health, and performance status could contribute to the higher rate of ARs [42,43]. In our retrospective study, patient comorbidities were not systematically assessed, and therefore, we were not able to further evaluate this question. 

We observed slightly inferior outcome parameters after transplantation in patients with AEs and patients ≥60 years of age, i.e., an elongated neutrophil and platelet recovery time, which is in line with other studies. In a study with patients suffering from multiple myeloma, a significantly prolonged platelet recovery time of 15 days was reported in patients ≥65 years, whereas patients <50 years only needed 13 days [44]. Likewise, Vaxman et al. reported a time of 15 days to reach neutrophil and platelet recovery (>0.5 × 10^9^/L; >20 × 10^9^/L) in a cohort of elderly patients (≥75 years), which is similar to 15 and 14 days in our cohorts of patients ≥60 years with and without AEs, respectively [26]. Among additional outcome parameters evaluated (fever later during the hospitalization, positive blood cultures, time in hospital), only the time in hospital was significantly longer in the group with AEs. Several factors such as age, disease, treatment, and patient management could influence the duration of hospitalization. Holbro et al. found that disease stage and age >60 years are risk factors for longer hospitalization in patients with multiple myeloma, and Jones et al. described longer hospital stays in patients conditioned with irradiation [45,46]. 

In our study, 25.8% of AEs were serious, requiring an immediate action to protect the patients or to minimize potential harm. Of note, more than half of AEs (57.5%) were categorized as potentially serious, indicating that deviations were immediately recognized, and measures were taken during the procedure before any harm could occur; however, this often came at the price of additional workload. Further, 16.7% of AEs were considered as non-serious and had only a minimal direct impact on the patient. Nevertheless, these AEs absorbed unnecessarily resources such as time of personnel and lead to increased costs. Therefore, prevention of non-serious and potentially serious AEs resulting from an inferior quality or procedure harbors great potential to improve cost efficiency. Such AEs could also contribute to ARs as well as to the outcome of the autoHSCT, e.g., lower numbers of CD34+ cells mobilized and collected, which leads to a higher infused volume and DMSO content [47]. 

Serious and potentially serious AEs classified in the categories quality made up 35.0% of total AEs. These were mainly due to issues concerning the transplant quality during collection and processing and a higher number of such AEs occurred in older patients. Most non-serious AEs were related to administrative issues and occurred in younger and older patients. Such AEs did not affect the patients directly, but they absorbed the time of personnel. Reducing and avoiding administrative errors and their corrections has therefore a great potential for time and cost savings. Based on our results, we propose that an increased focus should be laid on administrative, procedural, and qualitative aspects during autoHSCT to make substantial additional improvements and to further reduce the transplant-related risk especially also for older patients. Preventing potentially serious AEs of quality and procedural issues could already reduce the total number of AEs by 36.7%. To guarantee a high level of security for the patient, tight adherence to SOPs during the whole process of autoHSCT is important [48].

Computerized workflows covering all steps of cell collection, processing, cryopreservation, thawing, dispensing and administration allows to quality assure processes in real-time and greatly facilitate quality assurance checks and documentation of deviations that may occur during processing [49]. In our autoHSCT program, such an HSCT IT solution was developed and implemented several years ago, allowing us to control the sequential steps of the workflow that are conditional. To avoid critical mistakes, certain conditions must be met and approved by responsible individuals before proceeding to the next processing step. Hence, such electronic documentation and supervision of the entire HSCT workflow have a great potential to reduce AEs.

There are certain limitations regarding our study. Due to the retrospective nature, we cannot exclude that especially non-serious AEs might have been underreported. In addition, potential ARs due to the application of G-CSF might be lacking in our data set since it was a retrospective assessment and we could only evaluate AEs and ARs which were reported from the start of the cellular collection. Nevertheless, we identified some highly interesting correlations regarding age and occurrence of AEs. In the future, a more detailed analysis of various additional parameters could help to delineate and explain our findings more in depth. Validation of our results by prospective studies with a focus on specific parameters will certainly be of interest.

## 5. Conclusions

In summary, we found a relatively low number of AEs and only 6.0% of patients experienced ARs. Most of the AEs occurred during processing and transplantation, were potentially serious and were related to quality and procedural issues. Several factors such as higher age at transplantation, low CD34+ cell counts and large volumes collected during leukapheresis, large infusion volumes, and longer time for neutrophil recovery significantly correlated with the occurrence and number of AEs in our cohort. Importantly, patients over 60 years of age experienced significantly more AEs. Our results highlight steps and parameters for the potential optimization of the autoHSCT procedure, especially in elderly patients.

## Figures and Tables

**Table 1:** Characteristics of patients.

Patients	*n* = 449
Gender, *n* (%)	
• Male/Female	305 (68)/144 (32)
Age at 1st transplantation in years	
(median/range)	60/19–79
Diseases, *n* (%)	
• Plasma cell disorder	208 (46)
• Non-Hodgkin lymphoma	164 (37)
• Acute myeloid leukemia	44 (10)
• Other diseases	33 (7)

**Table 2:** Distribution of autoHSCT.

No. of Patients	No. of Separate Transplantations	Total No. of Transplantations (Thereof CD34+-Selections)	No. of Infusions per Transplantation
339	1	339 (80)	1
37	1	37	2
16	1	16	3
6	1	6	4
3	1	3	5
30	2	60 (2)	1
1	2	2	1 and 2
3	2	6	2
1	2	2	4 and 5
13	3	39	1
Total: 449		Total: 510	

**Table 3:** Collection, processing and transplantation characteristics. Data without the CD34+-selected transplants.

Variables	Total Transplantations *n* = 428	with AE *n* = 79	without AE *n* = 349	*p*
Collection and processing				
• Total volume collected [mL] ^1,2^	287/89–700	374/156–700	273/89–573	<0.0001 ****
• Lc in transplant product [×10^9^/L] ^1,2^	200/128–431	201/146–225	200/128–431	0.8990
• MNC in transplant product [×10^9^/L] ^1,2^	114/29–231	110/53–174	115/29–231	0.3189
• PNC in transplant product [×10^9^/L] ^1,2^	86/22–199	90/30–153	86/22–199	0.3041
• Total CD34+ cells in transplant product [×10^6^/kg BW] ^1^	8.7/2–29.6	6.2/2.1–18.4	9.6/2.0–29.6	<0.0001 ****
Transplantation				
• Age of patients at transplantation [years] ^1^	60/19–79	62/24–75	58/19–79	0.0013 **
• Volume infused [mL] ^1^	220/40–1680	310/70–1680	180/40–1440	<0.0001 ****
• Volume of DMSO infused [mL] ^1^	11.0/2.0–84.0	15.5/3.5–84.0	9.0/2.0–72.0	<0.0001 ****
• Neutrophil recovery [days] ^1,3,5^	11/1–32	12/8–20	11/1–32	0.0182 *
• Platelet recovery [days] ^1,4,6^	14/10–138	15/11–138	14/10–108	0.0120 *
• Body temperature ≥ 38.5 °C [yes/no/n.a. (% of total)]	401/25/2 (94)	71/7/1 (89)	330/18/1 (95)	‡ 0.20/0.19
• Duration of hospitalization [days] ^1^	21/1–71	22/1–71	21/1–65	0.0436 *

^1^: median/range; ^2^: at 1st apheresis; ^3^: patients who never reached the value were excluded, *n* = 3; ^4^: patients who never reached the value were excluded, *n* = 7; ^5^: >0.5 × 10^9^/L; ^6^: >20 × 10^9^/L; n.a. not available; ‡ Chi-square test and Fisher’s exact test; significance: * *p* ≤ 0.05, ** *p* ≤ 0.01, **** *p* ≤ 0.0001.

## Data Availability

The data presented in this study are available on request from the corresponding author.

## Supplementary Materials

The following supporting information can be downloaded at: https://www.mdpi.com/article/10.3390/cancers15051584/s1, Table S1: AEs; Table S2: Collection, processing, and transplantation characteristics according to reported AEs and age at transplantation; Table S3: Collection, processing, and transplantation characteristics according to the number of AEs reported at transplantation.
