# Supplementary material for: Higher Age (≥60 Years) Increases the Risk for Adverse Events during Autologous Hematopoietic Stem Cell Transplantation
Source: Cancers (Basel). 2023 Mar 3;15(5):1584. doi: 10.3390/cancers15051584 (PMC10000699; doi:10.3390/cancers15051584)
Supplement: Supplementary file 1 [file cancers-15-01584-s001.zip › cancers-2229981-supplementary.pdf]

Supplemental materials

Table S1

Table S1. AEs, n=120.

Subcategory	Reported AEs	n=	% of total AEs	Part of the process:		
				Collection	Processing	Transplantation
Administration	data/ planning related issue	11	9	1	9	1
	laboratory/ technical issue	1	1		1	
AR*	cardiovascular	9	8			9 (4)
	gastrointestinal	7	6			7 (3)
	allergic/ respiratory	7	6			7 (5)
	neurological	5	4			5 (2)
	fever	4	3			4
Material	damaged bag	8	7		8 (8)	
	laboratory/ technical issue	1	1		1 (1)	
Procedure	time delay	10	9			10 (4)
	data/ planning related issue	2	2		2	
	laboratory/ technical issue	1	1		1 (1)	
Quality	cell loss over limit	16	13		16 (1)	
	insufficient collection	14	12	14		
	positive initial bacterial/fungal culture, not confirmed	8	7	3	5	
	laboratory/ technical issue	6	5		6 (4)	
	data/ planning related issue	3	3	1	2	
Non-patient related	issues affecting several transplants such as					
	device failure	6	5			
	human error	4	3			
	material	1	1			

* 32 ARs in 28 patients; (number of serious AEs)

Supplemental materials
Table S2

Table S2. Collection, processing and transplantation characteristics according to reported AEs and age at transplantation. Data without the CD34+-selected transplants.

**Variables**	**Transplantations wAE<60 n=30**	**Transplantations wAE≥60 n=49**	**Transplantations woAE<60 n=183**	**Transplantations woAE≥60 n=166**
Collection and processing				
• Total volume collected [ml][1,2]	416.5/ 198-586	346/ 156-700	262.5/ 89-573	284/ 126-568
• Lc in transplant product [$\times 10^9$/L][1,2]	201.7/ 192.0-224.8	199.3/ 146.4-218.6	199.7/ 128.1-311.8	200.6/ 152.5-430.6
• MNC in transplant product [$\times 10^9$/L][1,2]	104.9/ 60.9-173.5	114.8/ 52.8-161.4	115.8/ 28.7-186.0	112.7/ 36.3-231.2
• PNC in transplant product [$\times 10^9$/L][1,2]	96.3/ 30.4-151.1	83.3/ 37.3-153.3	81.8/ 22.0-198.7	88.3/ 22.9-199.5
• Total CD34+ cells in transplant product [$\times 10^6$/kg BW][1]	7.5/ 2.1-13.7	5.7/ 2.1-18.4	10.9/ 2.0-29.6	7.1/ 1.5-21.5
Transplantation				
• Age of patients at transplantation [yrs][1]	54.5/ 24-59	67.0/ 60-75	49.0/ 19-59	65.0/ 60-79
• Volume infused [ml][1]	300/ 80-1650	320/ 70-1680	160/ 40-1380	240/ 40-1440
• Volume of DMSO infused [ml][1]	15.0/ 4.0-82.5	16.0/ 3.5-84.0	8.0/ 2.0-69.0	12.0/ 2.0-72.0
• Neutrophil recovery [days][1,3,5]	11/ 10-17	12/ 0-20	11/ 1-32	11/ 0-19
• Platelet recovery [days][1,4,6]	13/ 11-137	15/ 0-138	13/ 10-108	14/ 0-105
• Body temperature ≥38.5°C [yes/no/n.a. (% of total)]	26/ 3/ 1 (87)	45/ 4/ 0 (92)	172/ 10/ 1 (94)	158/ 8/ 0 (95)
• Duration of hospitalization [days][1]	19/ 16-34	23/ 1-71	20/ 1-65	22/ 1-48

1: median/ range; 2: at 1st apheresis; 3: patients who never reached the values were excluded, n=3; 4: patients who never reached the value were excluded, n=7; 5: >0.5 $\times 10^9$/L; 6: >20 $\times 10^9$/L; n.a. not available

Supplemental materials
Table S3

Table S3. Collection, processing and transplantation characteristics according to the number of AEs reported at transplantation. Data without the CD34+-selected transplants.

Variables	Transplantations woAE n=349	Transplantations w1AE n=63	Transplantations w≥2AE n=16
Collection and processing			
• Total volume collected [ml][1,2]	273/ 89-573	343.5/ 156-700	412.5/ 275-452
• Lc in transplant product [$\times 10^9$/L][1,2]	200.0/ 128.1-430.6	201.5/ 146.4-214.5	200.1/ 192.0-224.8
• MNC in transplant product [$\times 10^9$/L][1,2]	115.2/ 28.7-231.2	109.6/ 52.8-173.5	109.8/ 60.9-140.5
• PNC in transplant product [$\times 10^9$/L][1,2]	85.9/ 22.0-199.5	88.0/ 30.4-153.3	93.0/ 57.0-151.1
• Total CD34+ cells in transplant product [$\times 10^6$/kg BW][1]	9.6/ 1.5-29.6	6.5/ 2.1-18.4	5.1/ 2.1-9.8
Transplantation			
• Age of patients at transplantation [yrs][1]	58/ 19-79	62/ 30-75	65/ 24-71
• Volume infused [ml][1]	180/ 40-1440	300/ 70-1680	500/ 180-1650
• Volume of DMSO infused [ml][1]	9.0/ 2.0-72.0	15.0/ 3.5-84.0	25.0/ 9.0-82.5
• Neutrophil recovery [days][1,3,5]	11/ 0-32	11.5/ 0-20	12/ 11-20
• Platelet recovery [days][1,4,6]	14/ 0-10	14.5/ 0-60	24.5/ 11-138
• Body temperature ≥38.5°C [yes/no/n.a. (% of total)]	330/ 18/ 1 (95)	56/ 6/ 1 (89)	15/ 1/ 0 (94)
• Duration of hospitalization [days][1]	21/ 1-65	22/ 2-71	22/ 1-61

1: median/ range; 2: at 1st apheresis; 3: patients who never reached the values were excluded, n=3; 4: patients who never reached the value were excluded, n=7; 5: >0.5 $\times 10^9$/L; 6: >20 $\times 10^9$/L; n.a. not available
